# Inhibition of TGF-β by a novel PPAR-γ agonist, chrysin, salvages β-receptor stimulated myocardial injury in rats through MAPKs-dependent mechanism

**DOI:** 10.1186/s12986-015-0004-7

**Published:** 2015-03-09

**Authors:** Neha Rani, Saurabh Bharti, Jagriti Bhatia, Ameesha Tomar, T C Nag, Ruma Ray, Dharamvir Singh Arya

**Affiliations:** Department of Pharmacology, All India Institute of Medical Sciences, New Delhi, 110029 India; Department of Anatomy, All India Institute of Medical Sciences, New Delhi, 110029 India; Department of Pathology, All India Institute of Medical Sciences, New Delhi, 110029 India

**Keywords:** Chrysin, Isoproterenol, Myocardial injury, PPAR-γ, TGF-β, MAPKs

## Abstract

**Background:**

Pharmacological stimulation of peroxisome proliferator-activated receptor-gamma (PPAR-γ) has been recognized as a molecular switch in alleviating myocardial injury through modulating oxidative, inflammatory and apoptotic signaling pathways. This study was designed to elucidate the effect of chrysin, a novel PPAR-γ agonist and its functional interaction with TGF-β/MAPKs in isoproterenol-challenged myocardial injury in rats.

**Methods:**

Male Wistar Albino rats were either subjected to vehicle (1.5 mL/kg, p.o.) or chrysin (15–60 mg/kg, p.o.) for 28 days. Isoproterenol (85 mg/kg, s.c.) was administered to rats on 27^th^ and 28^th^ day to induce myocardial injury.

**Results:**

Chrysin dose dependently improved ventricular (±LVdP/*dtmax* and LVEDP) and hemodynamic (SAP, MAP and DAP) dysfunction in isoproterenol-insulted rats. This beneficial effect of chrysin was well supported with increased expression of PPAR-γ and decreased expression of TGF-β as evidenced by western blotting and immunohistochemistry analysis. Moreover, downstream signaling pathway of TGF-β viz. P-ERK½/ERK½ activation and P-JNK/JNK, P-p38/p38 and MMP-2 inhibition were also observed. Chrysin also attenuated NF-κBp65 and IKK-β expressions, TNF-α level and TUNEL positivity thereby validating its anti-inflammatory and anti-apoptotic properties. Additionally, chrysin in a dose dependent fashion improved NO level, redox status of the myocardium (GSH and MDA levels and SOD, GSHPx and CAT activities), cardiac injury markers (CK-MB and LDH levels) and oxidative DNA damage marker (8-OHdG level) and displayed preservation of subcellular and ultrastructural components.

**Conclusion:**

We established that activation of PPAR-γ and inhibition of TGF-β via MAPKs dependent mechanism is critical for cardioprotective effect of chrysin.

## Background

Peroxisome proliferator-activated receptor-gamma (PPAR-γ) is a transcription factor which apart from regulating glucose and lipid metabolism also controls cardiac metabolic hemostasis. Functionally, PPAR-γ stimulation plays a crucial role in controlling the expression of various genes involved in myocardial inflammatory and apoptotic signaling pathways. Moreover, cardiac PPAR-γ constitutively regulates redox hemostasis and is crucial in protecting cardiomyocytes from oxidative damage [1-3]. Additionally, PPAR-γ agonism was shown to increase cell survival in various models of myocardial injury [4]. Likewise, growing scientific evidence suggests that cross-talk between PPAR-γ and transforming growth factor-beta (TGF-β) regulates cardiomyocyte proliferation and differentiation [5-8]. Downstream pathways of TGF-β signaling including p38 mitogen-activated protein kinase (p38), extracellular signal-regulated kinase (ERK½), c-Jun N-terminal kinases (JNK) and matrix metalloproteinase-2 (MMP-2) was found to be significantly involved in cardiomyocyte injury, repair and remodeling and their pharmacological modulation have yielded significant outcomes in pre-clinical and clinical settings of various cardiovascular diseases including dilated cardiomyopathy, hypertrophy and myocardial infarction [8,9].

Interestingly, activation of PPAR-γ and simultaneously inhibition of TGF-β by various synthetic and phytopharmaceutical molecules was shown to abrogate the myocardial injury in rats. For instance, telmisartan and L-carnitine has been found to protect against arterial hypertension-related cardiac fibrosis and improve left ventricular remodeling in rats via activating PPAR-γ and inhibiting TGF-β signaling pathway [5,10]. Similarly, osthole, a phytopharmaceutical, has been reported to reduce isoprenaline-induced myocardial injury in mice via activating PPAR-γ and simultaneously inhibiting TGF-β expression [11]. In the same line of assumption we anticipated that chrysin (5,7-Dihydroxyflavone) a natural flavonoid obtained from honey (a highly nutritious food), propolis, and many fruits and vegetables could be of therapeutic interest as it possess PPAR-γ agonist activity [12]. Furthermore, the effect of chrysin on myocardial injury is still elusive. Accordingly, this study was designed to determine whether treatment with chrysin could improve the hemodynamic and ventricular dysfunction in isoproterenol-induced animal model of myocardial injury. Secondly, if so, could the activation of PPAR-γ and inhibition of TGF-β be the plausible mechanism in ameliorating isoproterenol-induced myocardial injury via modulating oxidative, apoptotic and inflammatory signaling pathways. Thus, for the first time we propose to evaluate the cardioprotective effects of chrysin based upon its effects on hemodynamic, biochemical, immunohistochemical, molecular, histopathological and electron microscopy.

## Materials and methods

### Animals

Male Wistar Albino Rats (4–6 weeks old, weighing 150–200 g) were approved and procured from Institutional Animal Ethics Committee of All India Institute of Medical Sciences, New Delhi, India (IAEC No. 716/13). All experiments were performed in accordance with the Indian National Science Academy Guidelines for the use and care of experimental animals. The rats were allowed free excess to standard pellet diet and tap water ad libitum and kept in polypropylene cages under relative humidity (60 ± 5%) and controlled temperature (25 ± 2°C) and subjected to light–dark cycle of 12:12 h.

### Reagents

Chrysin and isoproterenol was procured from Sigma Chemical Company (St. Louis, MO, USA) and was suspended in 0.5% carboxymethyl cellulose and dissolved in normal saline respectively. p44/42 MAPK (ERK½) (137 F5), phospho-p44/42 MAPK (ERK½) (Thr202/Tyr204), SAPK/JNK, phospho-SAPK/JNK (Thr183/Tyr185), TGF-β and IKK-β (L570) antibodies were purchased from Cell Signaling Technology, USA. PPAR-γ and P-p38 antibodies were purchased from Santa Cruz, USA and MMP-2, p38, β-actin, and NF-κBp65 antibodies were procured from Abcam Technologies, USA. Secondary antibodies were purchased from Merck GeNei, India. Creatine Kinase isoenzyme-MB (CK-MB) (Spinreact, Spain), 8-hydroxy-2′-deoxyguanosine (8-OHdG) (BMassay, Beijing, China), Rat Tumor necrosis factor-alpha (TNF-α) (Diaclone Tepnel Company, UK) and Lactate Dehydrogenase (LDH) isoenzyme (Logotech, Delhi, India) kits were used.

### Experimental protocol

Rats were divided into six groups with 10 animals in each group viz.

Group 1 (Sham): Rats were administered 0.5% carboxymethyl cellulose orally (1.5 mL/kg) for a period of 28 days. Consecutively, on 27^th^ and 28^th^ day the experimental animals were subcutaneously injected normal saline (1.5 mL/kg).

Group 2 (ISO): Rats were administered 0.5% carboxymethyl cellulose orally (1.5 mL/kg) for a period of 28 days. Consecutively, on 27^th^ and 28^th^ day the experimental animals were subcutaneously injected isoproterenol (85 mg/kg) to induce myocardial injury.

Groups 3–5 (Chr15, 30, 60 + ISO): Rats were administered chrysin (15, 30 and 60 mg/kg, p.o., respectively) for a period of 28 days. Consecutively, on 27^th^ and 28^th^ day the experimental animals were subcutaneously injected isoproterenol (85 mg/kg).

Group 6 (Chr60ps): Rats were administered chrysin (60 mg/kg, p.o., respectively) for a period of 28 days. Consecutively, on 27^th^ and 28^th^ day the experimental animals were subcutaneously injected normal saline (1.5 mL/kg).

### Induction of myocardial injury

Myocardial injury was carried out by injecting isoproterenol consecutively on 27^th^ and 28^th^ day of the protocol. On the 29^th^ day, rats were anesthetized with pentobarbitone sodium (60 mg/kg, i.p.) and a midline incision was given to open the chest. After 15 min of stabilization period, hemodynamic and left ventricular functions such as systolic arterial pressure (SAP), diastolic arterial pressure (DAP), mean arterial pressure (MAP), heart rate (HR), maximum speed of pressure development (±LVdP/*dtmax*) and the left ventricular end-diastolic pressure (LVEDP) were recorded using Biopac system software BSL 4.0 MP36. After completing the hemodynamic recordings, blood samples were withdrawn from the heart and the animals were sacrificed with an overdose of anesthesia (pentobarbitone sodium 100 mg/kg, i.v.). Their hearts were excised and processed for histopathological, ultrastructural, immunohistochemical, biochemical and molecular studies. The serum was separated via centrifugation (Heraeus Biofuge, Germany) at 3000g for 5 min.

### Biochemical studies

Ice-chilled phosphate buffer (0.1 M, pH 7.4) was used to prepare 10% heart homogenate and from that an aliquot was used for the estimation of Malondialdehyde (MDA) [13] and reduced Glutathione (GSH) levels [14]. In addition, supernatant obtained at 3000g for 20 min at 4°C was used to measure Lactate Dehydrogenase (LDH) and Nitrite levels (NO) [15], and Superoxide Dismutase (SOD) [16], Catalase (CAT) [17] and Glutathione Peroxidase (GSHPx) [18] activities. Furthermore, Creatine Kinase-MB (CK-MB) and Tumor Necrosis Factor-alpha (TNF-α) levels were measured spectrophotometrically in serum.

### Terminal deoxynucleotidyl transferase dutp nick End labeling (TUNEL) assay

In situ cell death detection kit, POD (Roche, Germany) was used to detect TUNEL positive cells following the manufacturer’s instructions.

### Histological and ultrastructural evaluation

Light and electron microscopic analysis of myocardial tissue was performed according to the method described in our previous study [19]. The pathologist performing histopathological and ultrastructural examination was blinded to the treatment protocol.

### Western blot analysis

According to the method described in our previous study [20], SDS-PAGE was used to separate heart tissues protein samples (40 μg), which were then transferred to nitrocellulose membrane (MDI, Ambala, India) and blocked for 2 h with 5% bovine serum albumin or non-fat dried milk. It was then incubated for 12 h at 4°C with primary antibody. The primary antibodies were detected with HRP-conjugated anti-rabbit/anti-mouse secondary antibody. The antibody-antigen complexes were visualized using enhanced chemiluminescence kit (Thermo scientific) under FluorChem M Protein imaging System (Bucher Biotec AG, Basel, Switzerland) and were quantified by Bio-Rad Quantity One 4.4.0 software (BIO-RAD, Hercules, CA, USA).

### Immunohistochemistry (IHC) analysis

VECTOR ABC KIT, CA, USA was used to perform IHC according to the method described in our previous study [20]. Briefly, slides were deparaffinized and hydrated through a series of xylene and graded alcohol. For antigen retrieval, slides were kept in pre-warmed citrate buffer (pH 6.0), washed 3 times for 5 minutes each in Tris Buffer Saline (TBS) and blocked for 45 minutes in ABC kit serum solution. After blocking, slides were then incubated overnight with primary antibody (PPAR-γ and TGF-β, 1:500 dilution) at 4°C. Moreover, slides were rinsed 3 times in TBS for 5 min and incubated in 3% H_2_O_2_ for 20 minutes to block the endogenous peroxidase activity. Slides were then washed 2 times with TBS and incubated for 45 minutes with secondary antibody (1:200 dilution) at room temperature. Slides were then again rinsed 3 times for 5 minutes with TBS and developed with 3,3′-diaminobenzidine. Slides were counterstained with haemotoxylin, mounted with DPX and visualized under microscope.

### Statistical analysis

The data were expressed as mean ± S.D. One way ANOVA followed by post hoc Bonferroni test was done using SPSS software 11.5. The value of P < 0.05 was considered as statistically significant.

## Results

### Effect of chrysin on hemodynamic and ventricular functions

**Figure 1:**
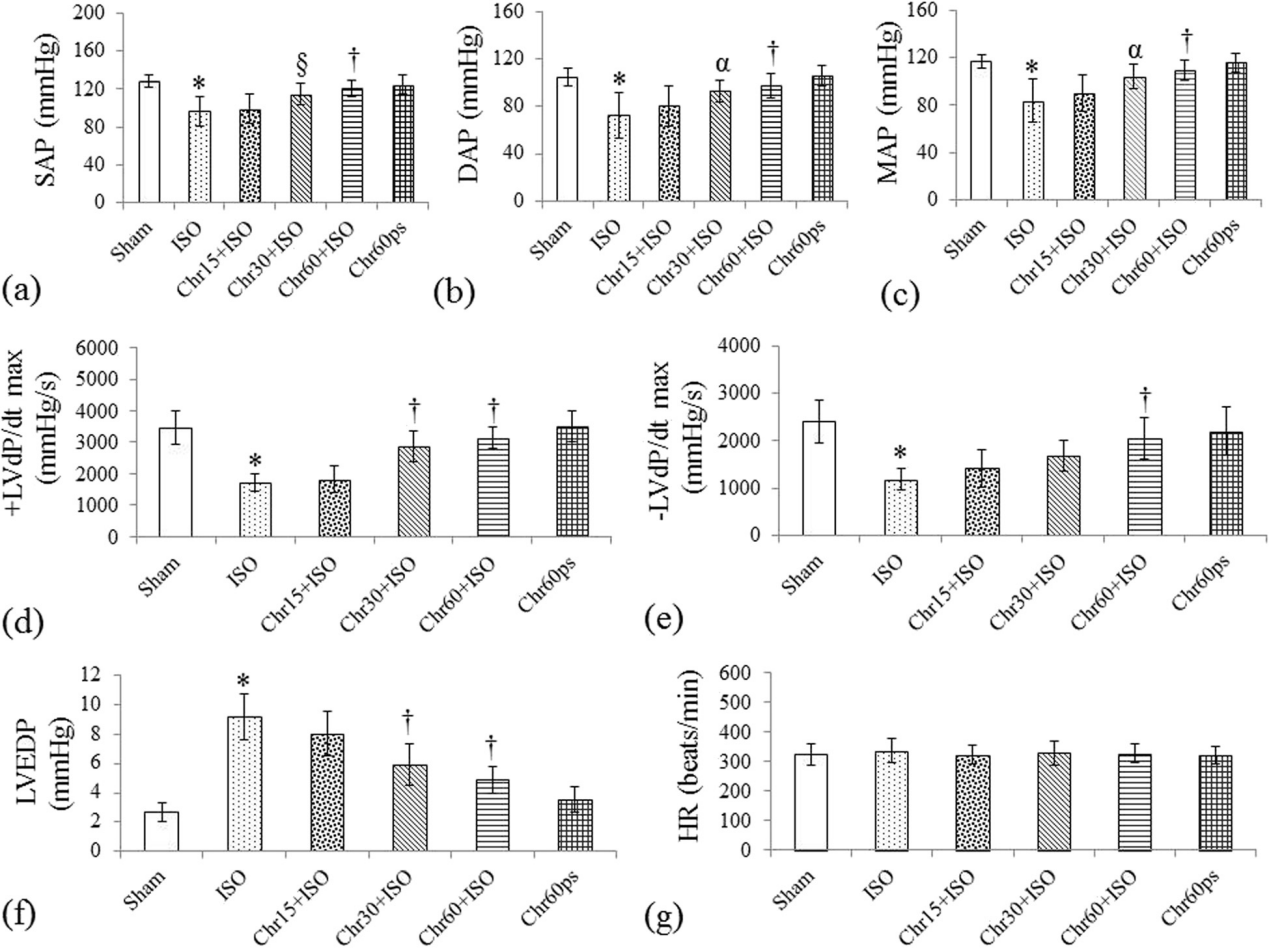
**Effect of chrysin on hemodynamic parameters following isoproterenol**-**induced myocardial injury. (a)** SAP: Systolic arterial pressure; **(b)** DAP: Diastolic arterial Pressure; **(c)** MAP: Mean arterial pressure; **(d)** + LVdP/*dtmax*: Maximal positive rate of left ventricular pressure; **(e)** -LVdP/*dtmax*: Maximal negative rate of left ventricular pressure; **(f)** LVEDP: Left ventricular end diastolic pressure and **(g)** HR: Heart rate. All values are expressed as mean ± S.D (n = 10/group).*P < 0.001 vs. sham and §P < 0.05, αP < 0.01, †P < 0.001 vs. ISO.

To investigate the ability of chrysin to alleviate cardiac functions we evaluated its effect on hemodynamic and ventricular assessments. Isoproterenol administration resulted in significant (P < 0.001) hemodynamic impairment in rats as observed through significantly reduced SAP, DAP and MAP as compared to sham group (Figure 1a-c). Similarly, significant (P < 0.001) ventricular dysfunction was also observed as exhibited through decreased contractility (+LVdP/*dtmax*), relaxation (−LVdP/*dtmax*) and increased LVEDP (Figure 1d-f). Interestingly, chrysin (15–60 mg/kg) dose dependently abolished the detrimental effect of isoproterenol and improved hemodynamic and ventricular dysfunction as observed by significant (P < 0.01) improvement in arterial pressures, ±LVdP/*dtmax* and LVEDP, though the level of significance (P < 0.001) was found to be greater with the highest dose (60 mg/kg) as compared to other two doses (Figure 1a-f). No significant change in HR was observed in any of the groups (Figure 1g).

### Effect of chrysin on various biochemical parameters

**Figure 2:**
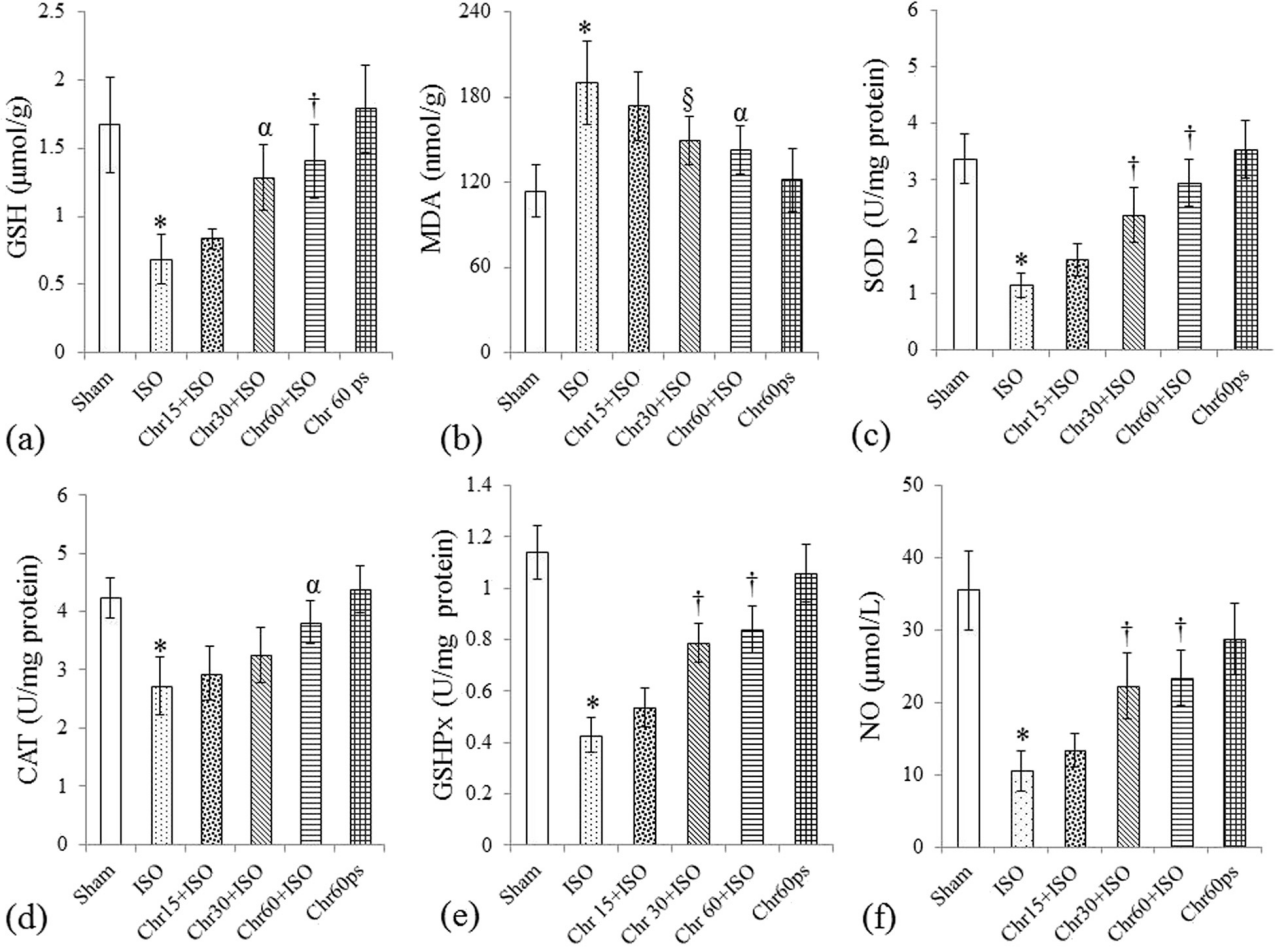
**Effect of chrysin on anti**-**oxidant parameters and NO level following isoproterenol**-**induced myocardial injury. (a)** GSH: Reduced glutathione; **(b)** MDA: Malondialdehyde; **(c)** SOD: Superoxide dismutase; **(d)** CAT: Catalase; **(e)** GSHPx: Glutathione peroxidase and **(f)** NO: Nitric oxide. All values are expressed as mean ± S.D (n = 6/group).*P < 0.001 vs. sham and §P < 0.05, αP < 0.01, †P < 0.001 vs. ISO.

**Figure 3:**
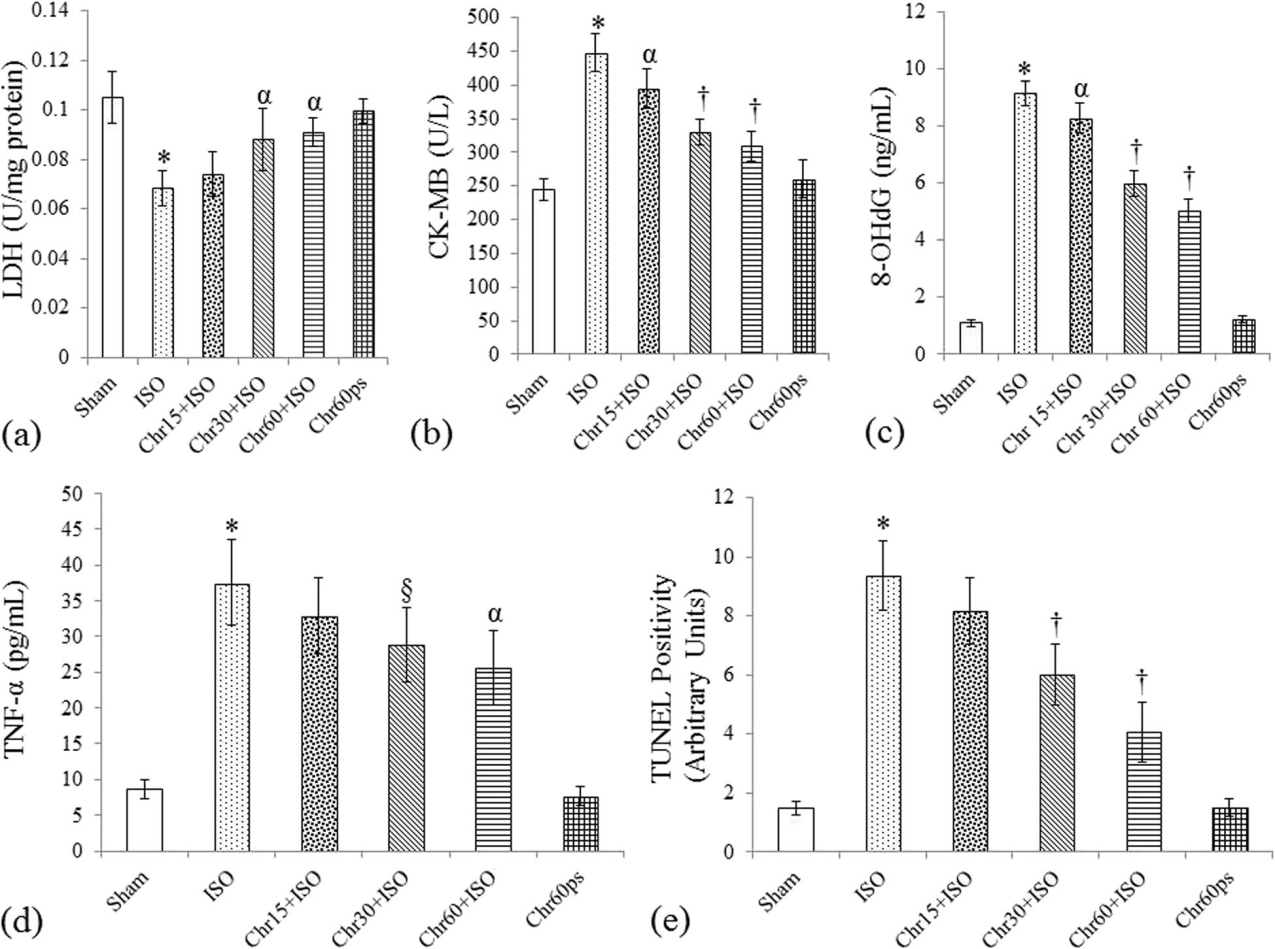
**Effect of chrysin on cardiac injury markers and 8**-**OHdG and TNF**-**α levels and TUNEL positivity following isoproterenol**-**induced myocardial injury. (a)** LDH: Lactate dehydrogenase; **(b)** CK-MB: Creatine Kinase-MB; **(c)** 8-OHdG: 8-hydroxy-2-deoxyguanosine; **(d)** TNF-α: Tumor necrosis factor-α and **(e)** Quantification of cardiomyocyte TUNEL positive nuclei. All values are expressed as mean ± S.D (n = 6/group).*P < 0.001 vs. sham and §P < 0.05, αP < 0.01, †P < 0.001 vs. ISO.

To further analyze the cardioprotective effect of chrysin, we assayed various oxidant-antioxidant proteins (GSH level and GSHPx, SOD and CAT activities), cardiac injury markers (CK-MB and LDH levels), oxidative DNA damage marker (8-OHdG level), MDA, NO and TNF-α levels. Myocardial injury induced by isoproterenol led to significant (P < 0.001) decrease in GSHPx, SOD and CAT activities and GSH, LDH and NO levels with concomitant increase in TNF-α, 8-OHdG, MDA and CK-MB levels, thus further strengthening the evidence for oxidative and inflammatory damage due to isoproterenol. Rats fed with chrysin (15–60 mg/kg) dose dependently normalized the above mentioned biochemical parameters though the effect was most pronounced (P < 0.01) at 60 mg/kg as compared to other two doses (Figures 2a-f and 3a-d).

### Effect of chrysin on various protein expression changes

**Figure 4:**
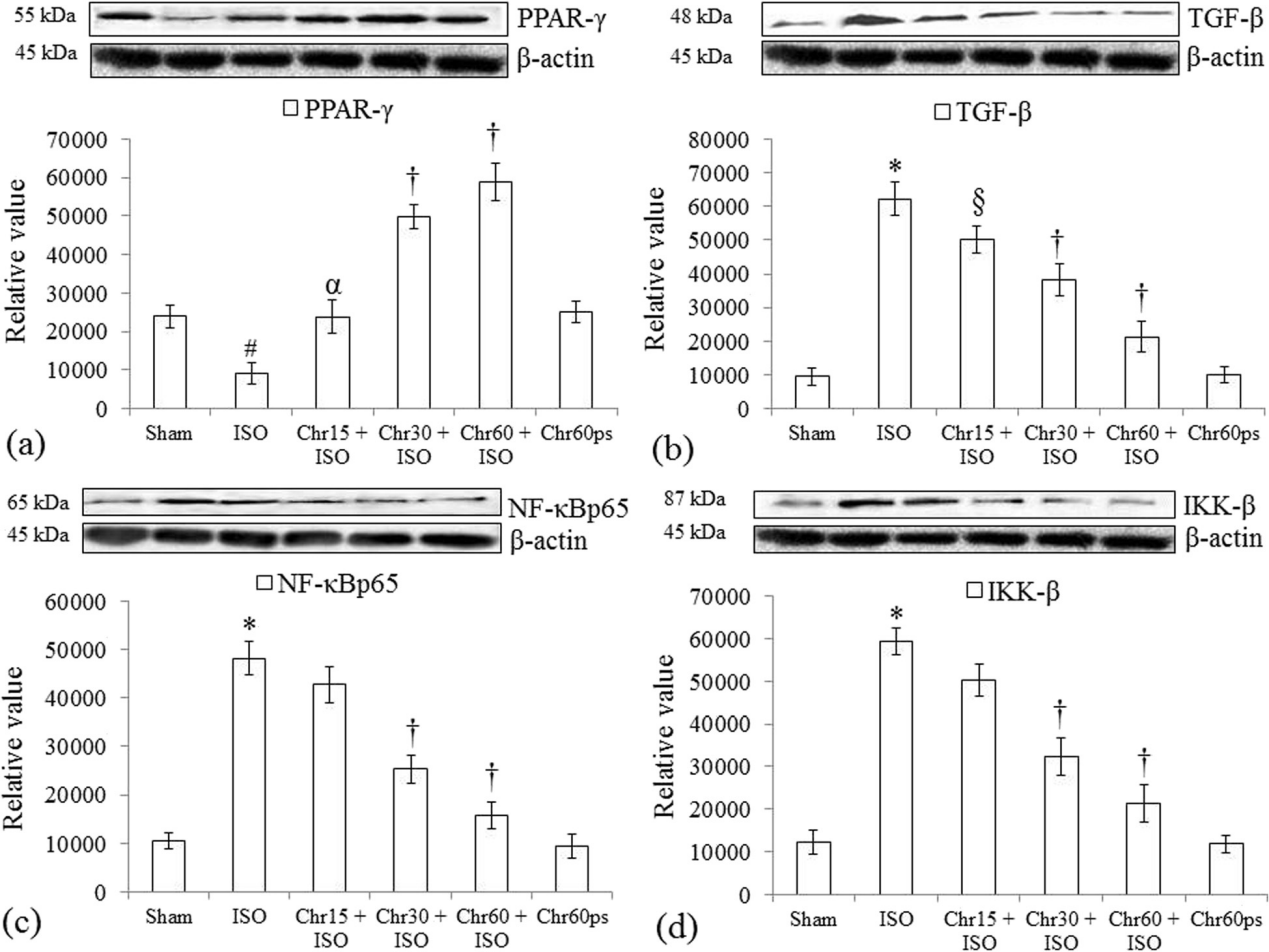
**Effect of chrysin on PPAR**-**γ,** **TGF**-**β and inflammatory protein expressions following isoproterenol-** **induced myocardial injury. (a)** PPAR-γ; **(b)** TGF-β; **(c)** NF-κBp65 and **(d)** IKK-β. All values for protein expressions are expressed as mean ± S.D (n = 3/group). #P < 0.01, *P < 0.001 vs. sham and §P < 0.05, αP < 0.01, †P < 0.001 vs. ISO.

To better understand the molecular role of chrysin in isoproterenol-insulted myocardium, we studied protein expression changes. Western blot analysis revealed that chrysin (15–60 mg/kg) dose dependently and significantly (P < 0.001) increased PPAR-γ and suppressed TGF-β protein expression as compared to isoproterenol group (Figure 4a and b).

Besides, to delineate the role of inflammation in our model, we assessed several inflammatory markers in heart. Western analysis revealed that chrysin mediated inhibition of inflammatory signaling in isoproterenol-induced myocardial injury is significantly (P < 0.001) linked to decreased NF-κBp65 and IKK-β protein expression in heart (Figure 4c and d).

**Figure 5:**
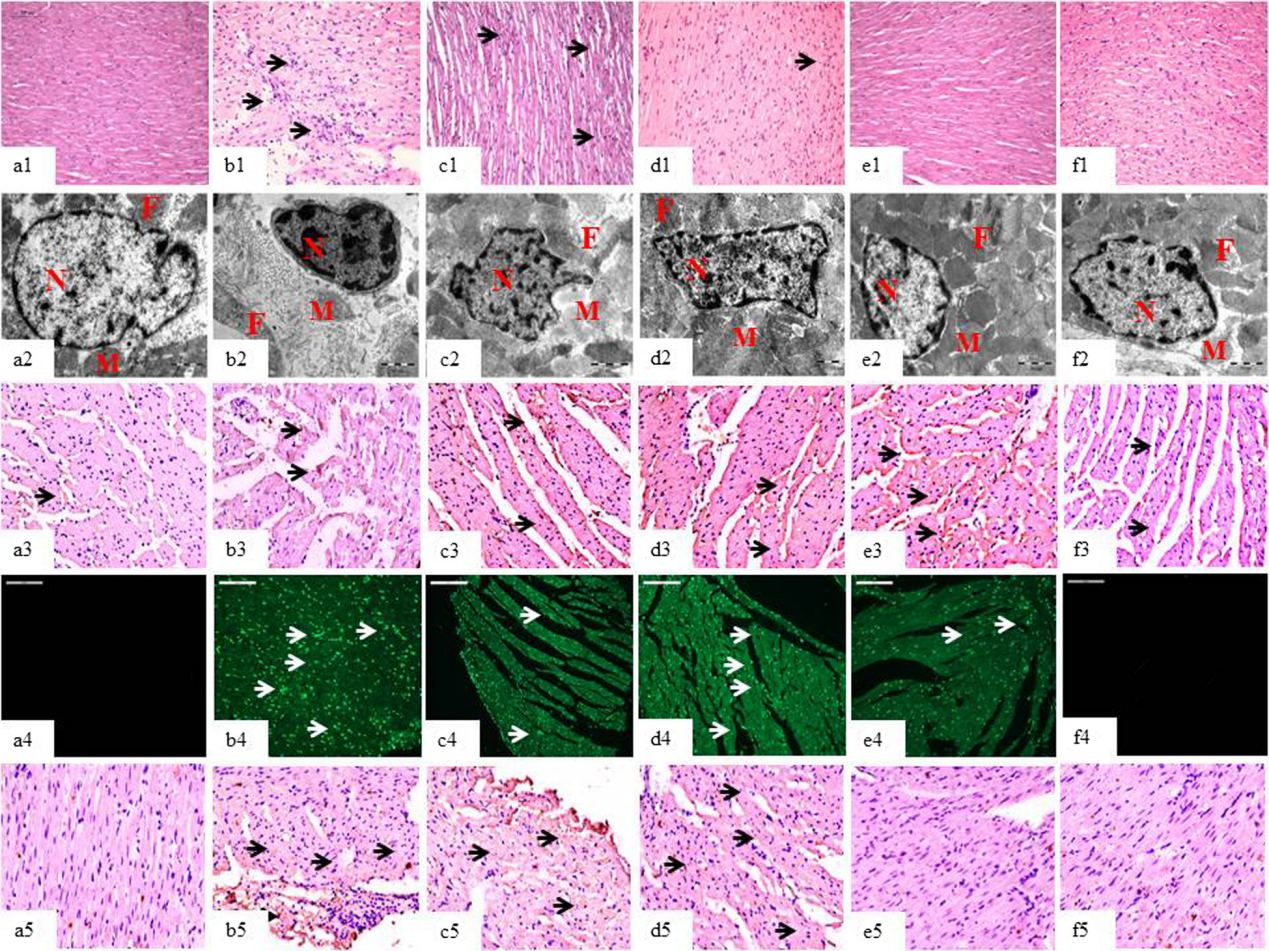
**Effect of chrysin on a1-** **f1:** **Light microscopic changes** **(10X,** **Scale bar 100 μm),** **a2-** **f2:** **Electron microscopic changes** **(4000X,** **Scale bar 1 μm,** **N:** **nucleus;** **MC:** **mitochondria;** **F:** **myofibrils),** **a3-** **f3:** **PPAR** **-γ immunohistochemistry** **(10X,** **Scale bar 50 μm),** **a4-** **f4:** **TUNEL positivity** **(20X,** **Scale bar 100 μm)** **and a5-** **f5:** **TGF-** **β immunohistochemistry** **(10X,** **Scale bar 50 μm)** **in different experimental groups.** Sham group **(a1-a5)**; ISO **(b1-b5)**; Chr15, 30, 60 + ISO mg/kg respectively **(c1-c5, d1-d5 and e1-e5)**; and Chr60ps **(f1-f5)**.

To further strengthen our western blotting findings, we performed immunohistochemistry analysis to check the distribution and localization of PPAR-γ and TGF-β within the myocardial cells. In consonance with western blotting results, we also found that chrysin significantly augmented PPAR-γ expression and mitigated TGF-β expression in recovered myocardium as compared to the failing myocardium (Figures 5a3-f3 and a5-f5).

**Figure 6:**
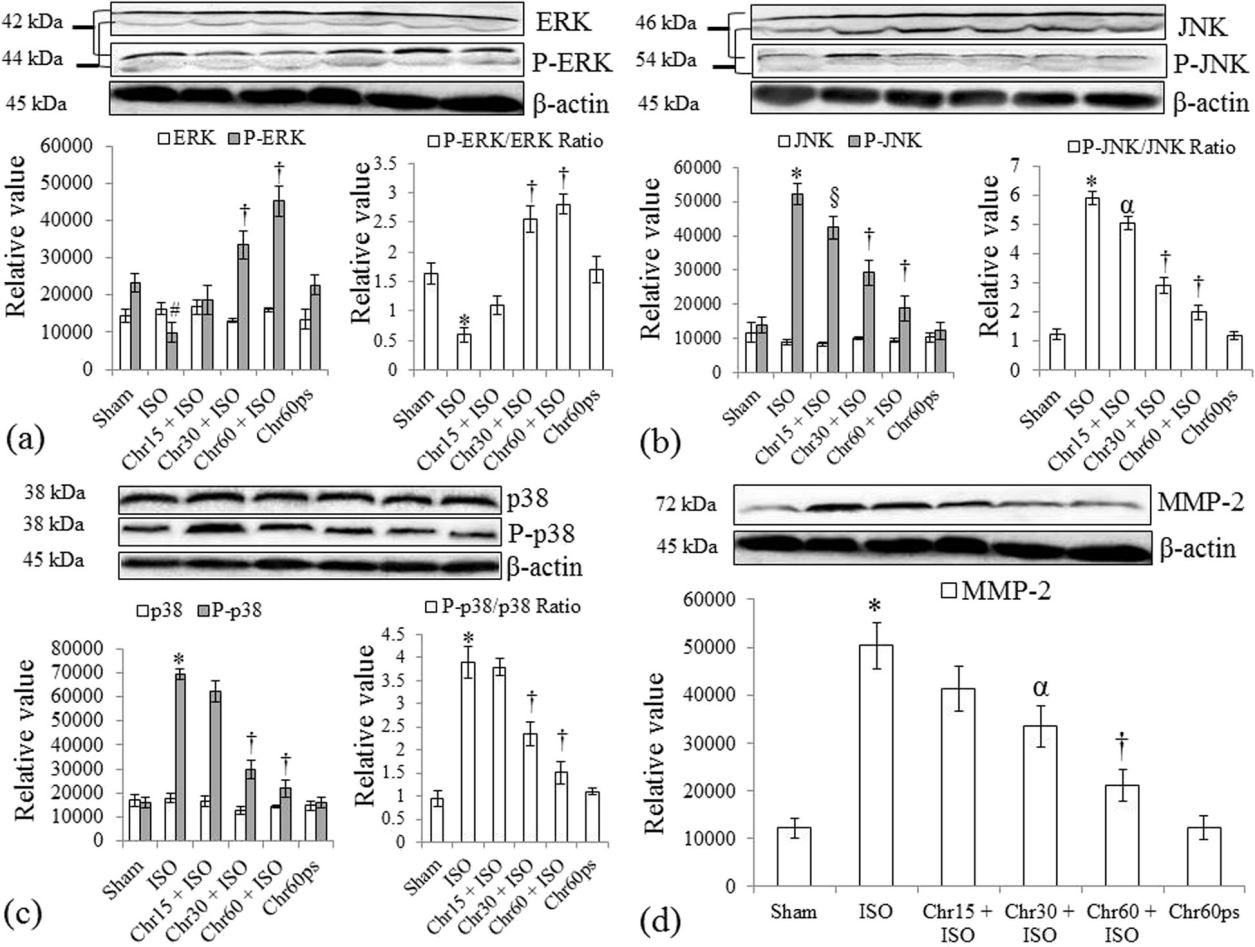
**Effect of chrysin on MAPKs protein expressions following isoproterenol**-**induced myocardial injury. (a)** ERK½ and P-ERK½; **(b)** JNK and P-JNK; **(c)** p38 and P-p38 and **(d)** MMP-2. All values for protein expressions are expressed as mean ± S.D (n = 3/group). #P < 0.01, *P < 0.001 vs. sham and §P < 0.05, †P < 0.001 vs. ISO.

Furthermore, to establish the potential role of chrysin on cell differentiation and survival, we studied protein expressions of MMP-2 and MAPKs pathway involving ERK½, P-ERK½, p38, P-p38, JNK, and P-JNK (Figure 6a-d). Intriguingly, we found that rats fed with chrysin augmented P-ERK½ to ERK½ protein expression ratio and attenuated P-p38 to p38 and P-JNK to JNK protein expression ratio and MMP-2 protein expression at 30 and 60 mg/kg but the effect was more significant (P < 0.001) at the highest dose following isoproterenol-induced myocardial injury (Figures 6a-d).

### Effect of chrysin on apoptosis

Next, we focused our interest on measuring the role of chrysin on apoptotic cell turnover in isoproterenol-challenged myocardium. To measure this, we performed TUNEL positivity assay as it allows immunohistochemical detection and quantification of apoptosis at single cell level based on specific labeling of nucleus DNA strand breaks. Chrysin dose dependently (P < 0.001) mitigated TUNEL positivity in isoproterenol treated rats, thereby validating a strong role of its anti-apoptotic property (Figures 3e and 5a4-f4).

### Effect of chrysin on histopathological and ultrastructural assessment

Figure 5a1 illustrates light micrograph features of sham group showing normal architecture of myocardium. In contrast, isoproterenol group showed extensive cardiomyocyte membrane damage with inflammatory cell infiltration, myonecrosis and marked edema (Figure 5b1 and Table 1). Chrysin (15–60 mg/kg) resulted in significant structural improvement as evidenced by decreased myonecrosis, edema and inflammatory cell infiltration in myocardium, the effect being most pronounced at 60 mg/kg (Figures 5c1-e1) and Table 1).

**Table 1:** **Effect of chrysin on histopathological grading**

**Treatment Groups**	**Myonecrosis**	**Inflammatory**	**Edema**
Sham	-	-	-
ISO	+++	+++	+++
Chr15 + ISO	+++	++	++
Chr30 + ISO	++	+	+
Chr60 + ISO	+	+	+
Chr60ps	-	-	-

Score (−): Absence of any myonecrosis, edema and inflammation; Score (+): Focal areas of myonecrosis, edema and inflammation; Score (++): Patchy areas of myonecrosis, edema and inflammation; Score (+++): Confluent areas of myonecrosis, edema and inflammation; Score (++++): Massive areas of myonecrosis, edema and inflammation (n = 6/group).

Figure 5a2 illustrates ultrastructural sections of sham group showing normal mitochondrial structure and myofibrils. Isoproterenol administration resulted in significant myofibrillar derangement, irregular mitochondria and chromatin condensation (Figure 5b2). Chrysin dose dependently improved ultrastructural components of the cardiomyocyte as the improvement was most pronounced in 60 mg/kg group (Figures 5c2-e2). The histopathological and ultrastructural changes in per se group (Figures 5f1 and f2) were similar to those found in sham group (Figures 5a1 and a2).

## Discussion

Pharmacological strategies targeted at activating PPAR-γ and suppressing TGF-β expression in pre-clinical studies have shown promising results in alleviating myocardial injury [5,6,10,11]. The results of the present study provide convincing evidence that oral administration of a novel compound, chrysin, exhibited a significant cardioprotective effect in isoproterenol-induced animal model of myocardial injury via PPAR-γ activation and TGF-β inhibition. The underlying mechanism behind this novel effect was primarily mediated through modulation of MAPKs and subsidence of apoptotic and inflammatory signaling pathway as observed via downregulation of TUNEL positivity and TNF-α/NF-κBp65/IKK-β expression respectively.

Catecholamines are known to regulate myocardial function. At a low dose, they exert inotropic effect and are beneficial, whereas at a high dose they produce deleterious effect on cardiac metabolism. Likewise, isoproterenol, a synthetic catecholamine and β-adrenergic agonist has been known to induce myocardial injury in rats. The myocardial damage produced by isoproterenol is irreversible in nature and occurs via free radical generation due to auto-oxidation and positive inotropic and chronotropic effect. Since hemodynamic, ventricular, biochemical, morphological, and histopathological changes following high dose isoproterenol administration in rats resemble closely to those occurring in patients with myocardial infarction, the isoproterenol-induced MI serves as a well-standardized model to study the beneficial effects and mechanism of many drugs [19,21-23]. As anticipated, in the present study, isoproterenol administered rats showed hemodynamic and ventricular dysfunction as evident by decreased contractility and relaxability and elevated preload as compared to sham group. These compromised functional abnormalities in heart were accompanied as well as substantiated with amplified necrosis, inflammatory cell infiltration and edema as observed on light and electron microscopical studies. Conversely, chrysin at the highest two doses (30 and 60 mg/kg) markedly improved the cardiac dysfunction and preserved the morphological architecture of the heart. The governing factors involved in improving hemodynamic status could be due to direct vasodilatory effect of chrysin via stimulating endothelial formation of NO and/or due to Na^+^-K^+^ pump activation perhaps through endothelium-derived hyperpolarizing factor [24-26]. Chrysin activates PPAR-γ receptors and it is well known that activation of PPAR-γ has a positive effect on cardiac metabolism and inhibition of cytosolic calcium overload [12,27]. Furthermore, modulation of downstream signaling pathways of TGF-β by chrysin viz. ERK½ activation and p-38/JNK/MMP-2 inhibition cannot be ruled out as a possible mechanism as these MAPKs plays a significant role in cardiomyocyte survival and demise [28,29]. Thus, the beneficial effect of chrysin on cardiac function is largely attributed through collective effect of activation of PPAR-γ and modulation of MAPKs.

Interplay between PPAR-γ, TGF-β and oxidative stress plays a crucial role in regulating myocardial injury. In the present study, isoproterenol-induced activation of oxidative stress has shown to modulate cardiac injury markers (CK-MB and LDH levels), attenuate PPAR-γ expression, reduce NO and GSH levels and GSHPx, CAT and SOD activities which were accompanied with amplified oxidative DNA damage marker (8-OHdG level), TGF-β expression and malondialdehyde level. Generation of free radicals by isoproterenol occurs via its quinine metabolites that react with oxygen to produce ROS, hydrogen peroxides and superoxide anions, which eventually consume and deplete the stores of endogenous antioxidants like GSH, GSHPx, SOD and catalase in myocardium. Also, malondialdehyde, a biomarker of oxidative stress and a product of the oxidative degradation of unsaturated fatty acids, is also augmented by isoproterenol. ROS so produced through these processes are toxic by-products of aerobic metabolism and are known to react extensively with cellular membrane and macromolecules thereby activating so called “Oxidative Machinery” in myocardium. Once activated, this machinery imbalances cardiac metabolism and hemostasis resulting in oxidative stress-induced myocyte demise [21-23,30]. Intriguingly, these biochemical and molecular changes were significantly normalized by chrysin in a dose dependent fashion as we observed improvement in redox status and NO level in the recovered myocardium. This was likely due to interaction of chrysin with the circulating free radicals produced during homeostatic processes and scavenging of superoxide, nitrosative, hydroxyl and lipid peroxyl radicals into non-harmful compounds as observed through amplification of intracellular GSH level and GSHPx, CAT and SOD activities. This correction may also be attributed to the direct antioxidant activity and scavenging properties of the hydroxyl groups in the 5^th^ and 7^th^ position of chrysin [31]. Additionally, PPAR-γ activation-mediated inhibition of oxidative stress by chrysin could also be one of the interesting mechanisms as it has shown to positively regulate myocardial energy metabolism and homeostasis via inhibiting ROS. Furthermore, direct PPAR-γ/ERK½ activation and TGF-β/p-38/JNK/MMP-2 inhibition has also shown to prevent the activation of NADPH oxidase and ROS production which could also be advocated as a potential protective mechanism of chrysin in limiting oxidative stress mediated myocardial injury. Moreover, this is in accordance with various other findings where chrysin has shown potent anti-oxidant effect in abrogating the cellular injury [31-35].

To further validate the antioxidant potential of chrysin, we assessed the effect of chrysin on 8-hydroxy-2-deoxy guanosine (8-OHdG), a product of oxidatively modified DNA base guanine and an established marker of degree of DNA oxidative damage. Increased level of 8-OHdG has found to be directly correlated in patients with heart failure and is one of the most common adducts formed by oxidative DNA damage by reactive oxygen species. In accordance with the previous studies [36-38], we also observed augmented level of 8-OHdG following myocardial damage. Chrysin in a dose dependent fashion significantly abrogated the increased 8-OHdG level which could be due to decreased ROS production via its antioxidant properties or upregulation of antioxidant enzymes. Similarly, several investigators have demonstrated the ability of chrysin to protect cellular damage and subsequent cell death [31-35].

Apart from improving the myocardial function and redox status of the myocardium, chrysin also showed significant contribution towards inhibiting inflammatory and apoptotic signaling pathways via antagonism of TNF-α/NF-κBp65/IKK-β and TUNEL positivity. This salubrious effect may be in part due to PPAR-γ activation by chrysin, as it is regarded as the master switch in controlling inflammation and its stimulation has been directly associated with inhibition of recruitment of inflammatory cytokines and suppression of NF-κBp65 and IKK-β protein expression [39,40]. Moreover, other plausible mechanism for its anti-inflammatory and anti-apoptotic response could be due to stimulation of ERK½ and/or inhibition of TGF-β/p-38/JNK/MMP-2 pathway as MAPKs has been regarded as one of the key regulator for cardiomyocyte apoptotic and inflammatory signaling pathway. In line with our findings, other studies have also established the role of chrysin as an anti-inflammatory and anti-apoptotic molecule [26-28,35,39,40].

## Conclusion

In view of the aforementioned findings, the relationship between chrysin-PPAR-γ-TGF-β seems to be correlative and demands subsequent experimental and clinical studies to fully realize its ability as a potent cardioprotective agent. Moreover, chrysin holds the potential as a novel phytopharmaceutical in ameliorating myocardial injury through inhibiting inflammatory and apoptotic signaling pathway and it could open many interesting avenues aimed at activating PPAR-γ or inhibiting TGF-β targeted therapeutics.
